# Delayed repair is the ideal management for posterior urethral injuries- FOR the motion

**DOI:** 10.4103/0970-1591.65414

**Published:** 2010

**Authors:** S. Joseph Philipraj

**Affiliations:** Departments of Urology and Surgery, Sikkim Manipal Institute of Medical sciences, 5th mile, Tadong, Gangtok, Sikkim- 737 102, India

**Keywords:** Delayed repair, immediate/early alignment, posterior urethral injury

## Abstract

Posterior urethral injuries are seen in trauma cases with pelvic fracture. The time-tested and honored method of management is immediate supra-pubic diversion followed by delayed repair. Immediate alignment as a management option is not new. It was abandoned 30 years ago due to high incidence of incontinence and impotence. However, of late there is a tendency towards immediate management of these injuries with various endoscopic maneuvers. Unfortunately, there is little evidence supporting this. Even these evidences are of limited in number and of limited duration of follow-up.

## INTRODUCTION

“It is the urologist who will have to share, with the patient, the burden of any residual urological disability when the thoracic, the abdominal, and even the orthopedic aspects are probably long forgotten” - Sir Richard Turner Warwick.[1] Mr. Richard Turner-Warwick[1] Posterior urethral injury complicates up to 10% of pelvic fractures arising from blunt pelvic trauma.[2] These injuries pose a significant management challenge. The morbidity and complications of urethral injury have been aptly described.[1]

The ultimate aim of therapy should be a continent and potent patient with no stricture.

Management options after acute trauma include supra-pubic tube placement with delayed reconstruction and early posterior realignment. The main controversy in the initial management of posterior urethral injuries is centered on isolated complete posterior urethral ruptures. Much water has flown down in the management of urethral injury with proponents of each modality claiming good results. However, it is universally accepted that supra-pubic cystostomy and delayed repair alone is the best management. In general the short-term success of immediate urethral realignment is excellent. But the long-term result is not rewarding.

## MANAGEMENT OF URETHRAL TRAUMA

The primary concern, in the patient with pelvic fracture urethral distraction injury is, Resuscitation of the patient to preserve life because of associated injuries, Divert urine away from the site of injury, Preserve the residual sphincter mechanism at the bladder neck, and Avoiding jeopardizing sexual function. All these aspects can be safely addressed by the time-tested and gold standard immediate suprapubic cystostomy (SPC) placement and delayed repair.[3] Another option available is immediate endoscopic alignment and surgical exploration urethral alignment over a catheter. Open surgery includes evacuation of the periurethral hematoma and realignment of the ruptured urethra over a catheter. But it is associated with problems and complications like excessive bleeding, added surgical trauma to the vascular and nerve supply which may in turn lead to severe strictures, and impotence.[2]

Currently, many papers have come out with the option of endoscopic urethral realignment.[4–10] But to do that we need, Appropriate operating room, Appropriate instruments, Appropriate patient, and most important of them all Appropriate surgeon who is very well-versed in the technique.

It is indeed difficult to have all these “Appropriate” situations all the time. The enthusiast of this approach claim the advantage of avoiding open reconstruction but add a rider provided that the procedure is successful. Unfortunately, when it is not successful, reconstruction is always delayed. The prolonged endoscopic realignment attempts also have the significant risk of infection of the pelvic hematoma.[4] When patients are treated with endoscopic realignment, the recurrent strictures which can be easily managed with urethroplasty end up having multiple endoscopic procedures. The much claimed result that stricture never develops following placement of a catheter across a urethral disruption injury rarely happens, but in most patients, mild stenosis 1 to 2 cm in length develops which requires further interventions.

### Techniques for primary alignment of posterior urethra

There are many techniques which are used for primary realignment.[4–11] These include:

- Simple passage of a catheter across the defect. This maneuver will be possible only in few cases and in partial ruptures only.
- Endoscopically assisted catheter realignment using flexible, rigid endoscopes and biplanar fluoroscopy.
- Use of interlocking sounds (‘railroading’) or magnetic catheters to place the catheter.[11]
- Pelvic hematoma evacuation and dissection of the prostatic apex (with or without suture anastomosis) over a catheter.
- Catheter traction or perineal traction sutures to pull the prostate back to its normal location.
- Traumatic posterior urethral injury and early realignment using magnetic urethral catheters.

The very fact that there are so many techniques available clearly demonstrates that no one technique is better and it is difficult to compare any of the techniques of realignment since there are no large series available in the literature and most of the studies are done on highly selected cases and randomization has not been done.

### Complications of posterior urethral injury

The entire discussion on the management of posterior urethral injury is centered on preventing/minimizing the incidence of complications. The complications are stricture, incontinence and impotence. Each method of intervention has its fair share of complications. However, it will be prudent to find an intervention which has the lowest rate of complications. There are many studies available comparing various techniques of urethral disruption management. We will analyze important studies available in the literature to come out with evidence-based conclusion regarding the optimal management option for posterior urethral injury.

### Stricture

The incidence of stricture varies with the type of management, being 100% with SPC diversion and delayed repair to 5-15% with other interventions. The cause of high rate of stricture in delayed management is obvious but when urethroplasty is done later the stricture is very much amenable with success rate being in the range of 90-95%.

Podestá *et al*,.[12] retrospectively reviewed the results of three types of initial management of pelvic fracture urethral disruption in children. They reviewed 35 boys from 1980 to 1994, age range from 2 to 15 years (mean age 8.1) in their study. Immediate treatment included, supra-pubic cystostomy and delayed urethroplasty (19 patients -Group 1), urethral catheter alignment without traction and concomitant supra-pubic cystostomy (10 patients -Group 2), and primary retro-pubic anastomotic urethroplasty (6 patients-Group 3).

In all patients in Groups 1 and 2 severe urethral obliteration developed even though they had catheter alignment. Four of Group 3 patients (66%) also developed a stricture at the site of anastomosis. The results clearly show that trying to align the urethra early does not prevent stricture formation but will end up having more interventions and ultimately if everything fails, open urethroplasty.

Elliott and Barrett[13] analyzed the long-term results of treatment of posterior urethral disruptions with immediate primary realignment. Fifty-six patients with complete urethral rupture were evaluated. Mean follow-up period was 10.5 years.

Thirty-six patients (68%) had post-realignment strictures which is quite high compared to delayed repair. Twenty-three (43.4%) were considered to have mild strictures but still required interventions.

Thirteen (24.5%) patients had more significant strictures that required a repeat procedure using general anesthesia.

A total of 20 procedures was required to treat the 13 patients.

Four patients required urethroplasty.

Hussman *et al*.[14] compared two modes of intervention. Sixty-four patients who suffered a prostatomembranous urethral disruption in association with a pelvic fracture were studied. Forty-seven patients were managed initially by supra-pubic cystostomy with delayed urethroplasty while 17 patients were managed initially by primary realignment. Notable finding was the requirement of secondary reconstructions for impassable strictures which developed in 53% of those treated by primary realignment. Only one patient in the latter group achieved urethral continuity that did not require further intervention. They concluded that primary realignment provides little in the way of long-term positive gains for the effort expended.

The short term and long term complications of immediate realignment are quite high compared to delayed management.

### Incontinence

Another complication of urethral injury is incontinence. There are many contributing factors like associated bladder neck injury. But isolated posterior urethral injury ideally should not have incontinence. The study by Podesta *et al*.[12] shows that after delayed urethroplasty 16 patients in Group 1 (84%) and all 10 in Group 2 were continent. But in spite of early alignment only three patients in Group 3 (50%) achieved continence. The result clearly shows that the early intervention may not prevent the problem.

One of the pioneers of urethral trauma surgery is Koriatim.[15] He analyzed data on pelvic fracture urethral injuries in the English literature for the last 50 years. One strong point of the review is that he included studies only if data were complete and conclusive. In the review the results clearly indicated that of the three conventional treatment methods primary suturing of the disrupted urethral ends had the greatest complication rates of incontinence and impotence (21 and 56%, respectively).

Primary realignment showed double the incidence of impotence and half that of stricture compared to supra-pubic cystostomy and delayed repair which was statistically significant. The results clearly show the advantages of delayed repair.

In a study done by Webster *et al*.[16] they compared two modalities of management. After doing meta-analysis they concluded that the incidence of incontinence was higher after primary alignment (20% versus 2%) compared to delayed repair.

### Erectile dysfunction

There are other studies which clearly demonstrate the disadvantages of higher incidence of erectile dysfunction and incontinence when compared to delayed reconstruction.[15–17]

The prominent among these studies is the one by Webster *et al*.[16] In this study the incidence of impotence was seen in 44% of patients treated with primary alignment compared with 11% of patients treated with delayed repair.

Feng *et al*.[18] studied the risk factors involved in the etiology of erectile dysfunction in men with urethral trauma. They evaluated 42 patients with traumatic urethral strictures secondary to blunt trauma. Eleven patients had organic erectile dysfunction demonstrated by nocturnal penile tumescence, vascular pathology was identified in only three patients. One important point which emerged from this study was that no significant difference could be observed in the end-to-end anastomosis procedure before and after surgery which was statistically significant, clearly showing that urethroplasty does not cause erectile dysfunction.

### Pediatric age group

Children could be different from adults in this respect. The lower urinary tract anatomy in children differs from that of adults, because the prostate is smaller. In addition, in prepubertal children, it is possible that the penile and spongiosal blood supply are tenuous compared with that of the adult with normal erectile activity; all these anatomical factors make children more prone to postoperative ischemic stricture formation. This is true especially when endourologic interventions are done repeatedly on these narrow small urethras. There are some studies depicting the early success of immediate/early interventions in children. However long-term results are not known. There are many studies showing very good results in children following delayed management.

Gundogdu *et al*.[19] studied 12 children with complete rupture of the posterior urethra who underwent primary alignment. Out of these 10 developed strictures and all these patients had problems of continence.

Orabi *et al*.[20] studied 50 boys with a mean age of nine years (6-13) with obliterative urethral stricture. Forty boys had undergone end-to-end anastomotic urethroplasty. With a mean follow-up of 4.5 years (Six months-seven years), all children who underwent perineal anastomotic urethroplasty were voiding well with no complications. This study clearly demonstrates advantages of delayed repair.

### Ejaculatory and fertility

One of the important long-term implications of ruptured urethra and its management is erection/ejaculation and fertility aspect. There aren’t many studies addressing this issue. It is obvious that more interventions on the urethra will definitely affect these aspects. Hence less intervention which is efficacious should reduce these problems. There are no studies addressing these issues after immediate/early management. Anger *et al*.[21] have found the ejaculatory profiles and fertility in men after posterior urethroplasty for pelvic fracture-urethral distraction defect injuries to be within normal limits in their review which clearly shows the advantage of delayed management over early intervention.

### Cost-effectiveness

Unfortunately, there are no studies available comparing the cost involved in the various types of management options available for complete isolated posterior urethral injury. It is obvious that endourological interventions will be more costly compared to open surgery. More interventions will also cost more. Hence a safer, simpler and effective mode of management is the answer and going by the evidence it is the delayed management which addresses all these issues.

### Long-term complications

There are many long-term complications of urethral injury and like urological, orthopedic, sexual, and most important the psychological complications. Not much data on long-term complications is available in the literature. However, one study by Abdurrahman Onen *et al*.[22] analyzed the long-term effects in children, especially the psychological outcome. They retrospectively reviewed the urethral injuries in boys from 1986-2000. The mean follow-up was 12 years (range 4 to 17).

The average age was eight years (range 3 to 13) at the time of trauma, and 20 years (range 8 to 32) at the

last follow-up visit. Most of the patients had urologic and orthopedic complications requiring more interventions. Psychiatric disorder was detected in 21 (42.9%) of the 49 patients. Significant contributing factors to the psychological problems were the number of urologic procedures required (more than three), presence of long-term complications, and total number of hospitalizations (more than three) secondary to the injury. Since immediate/early interventions do have significant complications which require repeated interventions and hospitalization, especially in children the long-term problems are to be expected. So a method of intervention which has less complications and intervention rate should be employed and going by the literature the delayed management of urethral injury should be the first line of management.

### Posterior urethral penetrating injuries

This is a very difficult entity to manage especially when the etiology is gunshot injuries. Literature is very sparse regarding this. Tausch *et al*.[23] have reviewed literature regarding this. In a retrospective review they studied 19 men who sustained posterior urethral gunshot injuries. Immediate primary repair was done only in two patients and delayed reconstruction in 15 patients. Two unfortunate patients ended up with prostatectomy. Of 15 patients who underwent delayed repair, 13 (86.6%) had normal flow rates and no lower urinary tract symptoms. The two remaining patients developed stricture recurrences and both were treated with open surgery and are doing fine. Two patients who underwent immediate primary repair had normal flow rates but it is difficult to make any concrete statement because the number is too low and it might have been very selective too. Their results clearly show that delayed repair following diversion gives better outcomes and also minimizes the number of subsequent interventions.

## CONCLUSION

As the incidence of trauma is increasing the incidence of pelvic fracture with posterior urethral injury is also increasing. Every case is to be judged individually to select the best options of available modalities. The standard intuitive approach dictates minimal early intervention with supra-pubic cystostomy which is certainly in concordance with the principles of damage control. Available evidence points towards supra-pubic cystostomy and delayed repair alone as the best initial management to prevent the patient of urethral injury from becoming a “urological cripple”.
